# Functional Outcomes Among Young People With Trajectories of Persistent Childhood Psychopathology

**DOI:** 10.1001/jamanetworkopen.2023.36520

**Published:** 2023-09-29

**Authors:** Niamh Dooley, Brendan Kennelly, Louise Arseneault, Stanley Zammit, Rob Whelan, Olivia Mosley, Delia Cotter, Mary Clarke, David R. Cotter, Ian Kelleher, Pat McGorry, Colm Healy, Mary Cannon

**Affiliations:** 1 Department of Psychiatry, Royal College of Surgeons in Ireland, Dublin, Ireland; 2 School of Business and Economics, University of Galway, Galway, Ireland; 3 Division of Psychological Medicine and Clinical Neurosciences, Cardiff University, Cardiff, United Kingdom; 4 Population Health Sciences, University of Bristol, Bristol, United Kingdom; 5 Trinity College Institute of Neuroscience, Trinity College Dublin, Dublin, Ireland; 6 School of Psychology, Trinity College Dublin, Dublin, Ireland; 7 Global Brain Health Institute, Trinity College Dublin, Dublin, Ireland; 8 School of Medicine, Royal College of Surgeons in Ireland, Dublin, Ireland; 9 Department of Health Psychology, Royal College of Surgeons in Ireland, Dublin, Ireland; 10 Centre for Clinical Brain Sciences, University of Edinburgh, Edinburgh, United Kingdom; 11 NHS Lothian Child and Adolescent Mental Health Service, Edinburgh, United Kingdom; 12 School of Medicine, University College Dublin, Dublin, Ireland; 13 University of Oulu, Faculty of Medicine, Oulu, Finland; 14 Social, Genetic and Developmental Psychology, King’s College London, London, United Kingdom; 15 Centre for Youth Mental Health, Orygen, Melbourne, Australia

## Abstract

**Importance:**

Understanding which children in the general population are at greatest risk of poor functional outcomes could improve early screening and intervention strategies.

**Objective:**

To investigate the odds of poor outcomes in emerging adulthood (ages 17 to 20 years) for children with different mental health trajectories at ages 9 to 13 years.

**Design, Setting, and Participants:**

Growing Up in Ireland is a longitudinal, nationally representative population-based cohort study. Data collection began in August 2007 and was repeated most recently in September 2018. All results were weighted to account for sampling bias and attrition and were adjusted for socioeconomic factors. Data analysis took place from October 2022 to April 2023.

**Exposure:**

Four latent classes captured variation in mental health in children aged 9 and 13 years, based on the parent-completed Strengths and Difficulties Questionnaire. Classes included no psychopathology, internalizing, externalizing, and high (comorbid) psychopathology. Those who remained in the same class from ages 9 to 13 years were included.

**Main Outcomes and Measures:**

Poor functional outcomes in emerging adulthood were measured at approximate ages 17 years (range, 16 to 18 years) and 20 years (range, 19 to 21 years). Outcomes included poor mental health, poor physical health, social isolation, heavy substance use, frequent health service use, poor subjective well-being, and adverse educational/economic outcomes.

**Results:**

Of 5141 included participants, 2618 (50.9%) were male. A total of 3726 (72.5%) were classed as having no childhood psychopathology, 1025 (19.9%) as having persistent externalizing psychopathology, 243 (4.7%) as having persistent internalizing psychopathology, and 147 (2.9%) as having persistent high psychopathology. Having any childhood psychopathology was associated with poorer functional outcomes in emerging adulthood. The internalizing group had elevated odds of most outcomes except for heavy substance use (range of odds ratios [ORs]: 1.38 [95% CI, 1.05-1.81] for frequent health service use to 3.08 [95% CI, 2.33-4.08] for poor mental health). The externalizing group had significantly elevated odds of all outcomes, albeit with relatively small effect sizes (range of ORs: 1.38 [95% CI, 1.19-1.60] for frequent health service use to 1.98 [95% CI, 1.67-2.35] for adverse educational/economic outcomes). The high psychopathology group had elevated odds of all outcomes (nonsignificantly for frequent health service use), though with wide confidence intervals (range of ORs: 1.53 [95% CI, 1.06-2.21] for poor physical health to 2.91 [95% CI, 2.05-4.12] for poor mental health). Female participants with any psychopathology had significantly higher odds of poor physical health and frequent health service use compared with male participants with any psychopathology.

**Conclusions and Relevance:**

In this longitudinal cohort study, childhood psychopathology was associated with a widespread pattern of functional impairment in emerging adulthood. Findings point to the need for a wider range of preventive interventions in child and adolescent mental health services.

## Key Points

**Question:**

What functional outcomes in emerging adulthood (ages 17 to 20 years) are associated with persistent childhood psychopathology (ages 9 to 13 years)?

**Findings:**

In this cohort study of 5141 participants, all types of psychopathology in childhood (internalizing, externalizing, or both) were significantly associated with poor functioning in emerging adulthood. This included poor mental and physical health, social isolation, heavy substance use, frequent health service use, poor subjective well-being, and adverse educational/economic outcomes.

**Meaning:**

These findings highlight the lasting effects of childhood psychopathology on functional outcomes in emerging adulthood and point to the need for a public health approach to youth mental health.

## Teaser

This cohort study evaluates odds of poor outcomes in emerging adulthood (ages 17 to 20 years) for children with different mental health trajectories at ages 9 to 13 years.

## Introduction

Children with mental health problems are at increased risk of an array of difficulties in young adulthood. Previous research has focused on adult outcomes of specific child and adolescent diagnoses, such as attention-deficit/hyperactivity disorder (ADHD)^1,2^ and depression,^3^ and on children who engaged with mental health services.^4^ However, children with subthreshold symptoms can also be at increased risk of poor functional outcomes,^5,6^ and only half of adolescents with a probable mental disorder receive professional support.^7,8^ It is therefore important to identify which children in the general population are at risk of poor functional outcomes, regardless of their diagnostic status or help-seeking behavior. Studies have shown that children with psychopathology who receive psychological/psychiatric treatment often have better long-term functional outcomes than those who do not receive treatment.^9,10,11,12^

Childhood psychopathology is associated with numerous functional impairments in adulthood, including mental disorder in adulthood.^1,3,4,13,14,15,16^ Childhood psychopathology has been linked with subsequent respiratory problems, infectious disease, cardiovascular disease, and weight problems.^17,18,19^ This may be partially mediated by smoking, alcohol use disorder, and illicit drug use.^1,3,20,21^ Social isolation and loneliness in young adulthood are also linked with childhood psychopathology, even after accounting for social isolation in childhood.^22,23^

Childhood psychopathology also has long-term economic costs. It is associated with being not in employment, education, or training (NEET) and claiming social benefits in adulthood,^4,24,25,26^ which may be partially explained by lower educational attainment.^26,27,28,29^ Childhood psychopathology may also be associated with more frequent health service use in adulthood; however, this has only been prospectively investigated for childhood ADHD, for example.^1^

**Figure 1.**
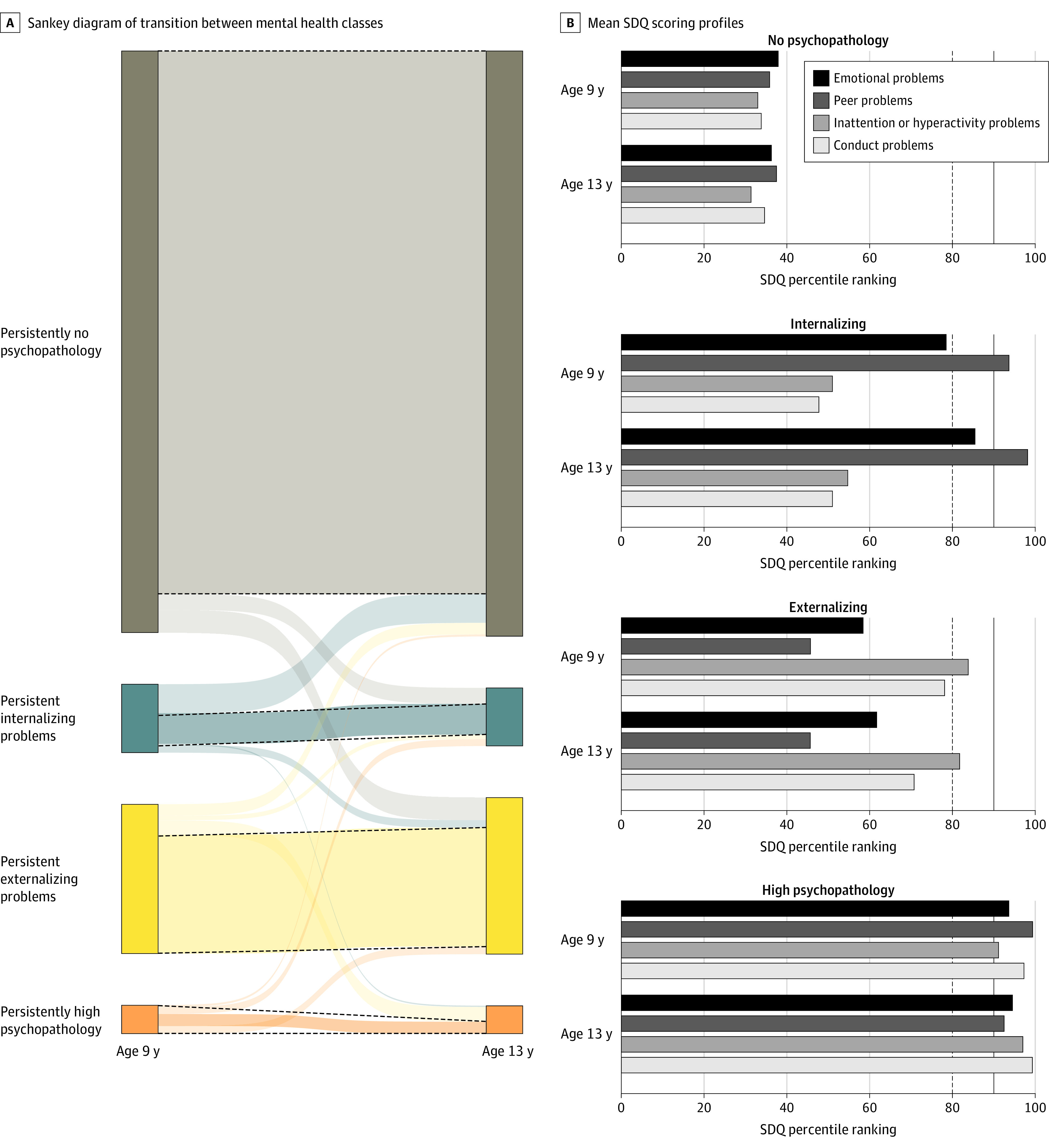
Characteristics of Childhood Groups A, Sankey diagram, adapted from Healy et al,^30^ showing the transition between mental health classes at ages 9 and 13 years. The dotted lines indicate the subsamples chosen for this study. B, Mean Strengths and Difficulties Questionnaire (SDQ) scoring profiles for each class, split by age. The vertical dotted line indicates the 80th percentile; the vertical bold line, the 90th percentile.

We opted to group children by their mental health symptoms over time, using data-driven methods. We did so because (1) childhood is characterized by significant movement between diagnostic categories^16^ and (2) diagnostic thresholds are relatively arbitrary.^6^ Previously, we performed latent class transition analysis on the Growing Up in Ireland longitudinal cohort.^30^ Childhood psychopathology groups were based on longitudinal patterns of scores from the Strengths and Difficulties Questionnaire (SDQ) across ages 9, 13, and 17 years. Four groups were identified and were characterized by low scores in all SDQ domains (no psychopathology), moderate to high conduct and hyperactivity problems (externalizing psychopathology), high emotional and peer problems (internalizing psychopathology), and high scores in all domains (high psychopathology) (Figure 1). In this study, we focused on individuals who remained in the same group between ages 9 and 13 years, which captures most children (85%) and is more likely to capture significant psychopathology.

In these groups, we investigated 7 areas of functioning in emerging adulthood: mental health, physical health, heavy substance use, social isolation, health service use, subjective well-being, and educational/economic outcomes. We hypothesized that those in the childhood psychopathology groups (internalizing, externalizing, and high psychopathology) would have more poor functional outcomes compared with the no psychopathology group.

## Methods

### Participants

Growing Up in Ireland is an ongoing longitudinal study of children in Ireland. It was commissioned by the Irish government and funded by the Department of Health and Children, the Department of Social and Family Affairs, and the Central Statistics Office. Participants were originally sampled from primary schools nationwide and constituted a nationally representative sample of 9-year-olds in Ireland (N = 8658).^31^ This sample was followed-up at ages 13 years (n = 7423; 88% retention), 17 years (n = 6216; 74% retention), and 20 years (n = 5190; 61% retention). The latter 2 waves included participants from a range of ages from 16 to 18 years (80% aged 17 years) and 19 to 21 years (91% aged 20 years), respectively. For simplicity, we refer to these as the age 17 years and age 20 years time points. Given the continuity of age ranges, we combined information from these 2 waves to form outcome measures during emerging adulthood (ages 16 to 21 years). Further sampling detail is available in eMethods 2 in Supplement 1, and a flowchart of the sample size is shown in eFigure 1 in Supplement 1.

Growing Up in Ireland received ethical approval from the Health Research Board of Ireland. Informed assent and consent were provided by participants and their parents/carers, respectively. This study followed the Strengthening the Reporting of Observational Studies in Epidemiology (STROBE) reporting guideline.

### Measures

#### Childhood Psychopathology

In a previous study on this cohort,^30^ latent class analysis and LCTA were applied to the 4 subscales of the parent-reported SDQ scores (emotional, peer, hyperactivity/inattention, and conduct problems) from ages 9 years, 13 years, and 17 years (n = 6039). Both analyses suggested a 4-class model was the best fit to the data. Individuals were ascribed to a group based on posterior probabilities, with probabilities of final group membership ranging from 55% to 95%. Model entropy scores suggested low levels of misclassification.

Figure 1A depicts the 4 groups and intergroup transitions between ages 9 and 13 years. Only those in the same LCTA group at both 9 and 13 years were included in this study. Figure 1B shows mean SDQ percentile rankings for each group at ages 9 and 13 years (raw SDQ score averages in eTable 3 in Supplement 1). For descriptive purposes, we consider a score of 16 or more on the SDQ total problems scale as high psychopathology, indicating clinical significance.

#### Emerging Adult Outcomes

Thirty dichotomous variables reflecting various functional outcomes were extracted from data at age 17 years and age 20 years. These variables were grouped into 7 categories, which were the primary outcomes: poor mental health, poor physical health, heavy substance use, frequent health service use, social isolation, poor subjective well-being, and adverse educational/economic outcomes. Poor mental health in emerging adulthood was defined as the presence of a mental health difficulty or at least 1 consultation with a mental health professional in the past year at either ages 17 years or 20 years. Criteria for poor physical health included obesity, difficulties sleeping, or poor general health at ages 17 years or 20 years. Heavy substance use was defined as daily cigarette smoking or alcohol use disorder (scores of 15 or more on the Alcohol Use Disorders Identification Test questionnaire) reported at ages 17 years or 20 years. Frequent health service use was defined as at least 1 visit to the accident and emergency department of a hospital or more than 5 visits to their general practitioner per year at ages 17 years or 20 years. Social isolation was considered as having fewer than 3 friends or having nobody to turn to for help and advice at ages 17 years or 20 years. Adverse educational/economic outcomes included low educational attainment (300 or less points in the Leaving Certificate examinations), being NEET, claiming social welfare, or reporting difficulty making ends meet at age 20 years. All outcome variables were self-reported by the participant except mental health difficulties and general health at age 17 years (eTable 1 in Supplement 1). Almost all participants had data on mental health, physical health, substance use, and health service use in emerging adulthood (range, 5140 to 5141); 5117 to 5118 had data on social isolation and subjective well-being; and 4140 had data on educational/economic outcomes (eFigure 1 in Supplement 1).

#### Covariates

Covariates included sex (male or female) and 3 socioeconomic factors measured at age 13 years: household income, parental education, and single parenthood. Household disposable income was equivalized to account for differences in the size and composition of families and split into deciles to avoid outliers. Parental education was measured by the highest education level among both parents.

### Statistical Analysis

All analyses were performed using R version 4.2.2 (The R Foundation). Logistic regressions were used to estimate the odds of each of the 7 primary outcomes for any persistent psychopathology in childhood (internalizing, externalizing, or high psychopathology) and for each psychopathology group in reference to the no psychopathology group. We also compared the odds of each outcome in a pairwise fashion across the 3 psychopathology groups.

A 2-tailed Bonferroni-corrected threshold of .007 (.05/7) was used to indicate significance. All reported estimates were fully adjusted for sex and socioeconomic factors. Case weights were used in all analyses to ensure representativeness of the target population and to account for attrition. Age 20 years weights were used where available (n = 4024) and age 17 years weights used for all others (n = 1117). Weights were renormalized such that their sum equaled the total sample size. Uncorrected χ^2^ and analysis of variance tests were used to determine whether differences in demographic and clinical variables were significant across groups.

The 7 outcome categories were the primary outcomes and represented any indicator of that particular functional impairment at age 17 years or 20 years. However, we also report adjusted odds of each specific indicator to illustrate how associations may vary across outcome subtype (eg, obesity vs sleep problems). To check whether results differed between male and female participants or between outcomes measured at age 17 years and age 20 years, we performed sex-stratified and age-stratified analyses.

To triangulate methods and facilitate replication, we performed additional analyses using more traditional cutoff points on the SDQ to define child psychopathology. We used the larger sample of 6039 participants (before removing those who changed class) to create these groups. High psychopathology was defined by scores in the 80th percentile or higher for all 4 SDQ scales at ages 9 years and 13 years. Internalizing psychopathology was defined as scoring in the 80th percentile or higher in either peer or emotional problems at ages 9 years and 13 years (lower than 80th percentile on the other scales). Externalizing psychopathology was defined as scoring in the 80th percentile or higher in either hyperactivity or conduct problems at both ages 9 years and 13 years (lower than 80th percentile on other scales). No psychopathology was defined by scoring lower than the 80th percentile in all SDQ scales at both ages.^32,33^ Further methodological detail is provided in eMethods 1 and 2, eTables 1 to 3, and eFigures 1 and 2 in Supplement 1.

## Results

Of 5141 included participants, 2618 (50.9%) were male. A total of 3726 (72.5%) were classed as having no childhood psychopathology, 1025 (19.9%) as having persistent externalizing psychopathology, 243 (4.7%) as having persistent internalizing psychopathology, and 147 (2.9%) as having persistent high psychopathology (Figure 1A). Age at outcome ranged from 16 to 21 years, and rates of high SDQ total scores ranged from 4% to 5%. Childhood psychopathology groups differed on the proportion of male to female participants and socioeconomic factors, validating the inclusion of these covariates in adjusted models (Table 1).

**Table 1.** Demographic and Clinical Characteristics of the Sample

Characteristic	Participants, No. (%)					Group difference^a^^,^^b^
	Full sample	Persistent psychopathology groups				
		None	Externalizing	Internalizing	High	
Total, No.	5141	3726	1025	243	147	NA
Sex						
Female	2523 (49.1)	1887 (50.6)	450 (43.9)	121 (49.6)	65 (44.4)	16.2^c^
Male	2618 (50.9)	1838 (49.4)	576 (56.1)	123 (50.4)	82 (55.6)	
Age at outcome (wave 3)						
Age 16/17 y^d^	4124 (80.2)	2979 (80.0)	835 (81.5)	193 (79.3)	116 (79.3)	1.4
Age 18 y	1017 (19.8)	746 (20.0)	190 (18.5)	49 (20.7)	30 (20.7)	1.4
Age at outcome (wave 4)						
Age 19/20 y^d^	3694 (91.8)	2706 (92.3)	718 (91.0)	181 (87.4)	89 (91.1)	7.0
Age 21 y	330 (8.2)	225 (7.7)	71 (9.0)	26 (12.6)	9 (8.9)	7.0
Low parental education^e^^,^^f^	661 (12.9)	404 (10.9)	161 (15.7)	35 (14.5)	60 (41.0)	125.0^g^
Single parent home^f^	875 (17.0)	513 (13.8)	249 (24.3)	66 (27.2)	47 (32.1)	107.5^g^
Income decile, mean (SD)^f^^,^^h^	6.21 (2.86)	6.40 (2.85)	5.66 (2.82)	5.66 (2.62)	4.43 (2.58)	36.1^g^
High SDQ total problem score at age 9 y^i^	241 (4.7)	0	85 (8.3)	26 (10.6)	130 (90.9)	2602.6^g^
High SDQ total problem score at age 13 y^i^	211 (4.1)	0	38 (3.7)	38 (15.5)	135 (92.2)	3138.0^g^

Abbreviations: NA, not applicable; SDQ, Strengths and Difficulties Questionnaire.

^a^ Group differences across persistent psychopathology groups refer to χ^2^ statistics or *F* values from analyses of variance for differences in frequencies and means, respectively.

^b^ All statistics are weighted to account for sociodemographic sampling bias and attrition.

^c^ *P* < .01.

^d^ Too few participants aged 16 and 19 years to list separately (less than 30 in full sample).

^e^ Low parental education defined as participants with a parent(s) who did not complete Leaving Certificate (final secondary school examinations) or an equivalent.

^f^ Recorded at participant age 13 years.

^g^ *P* < .001.

^h^ Deciles reflect the equivalized disposable income of the household, split into 10 roughly equal groups (eMethods 1 in Supplement 1).

^i^ High SDQ scores defined as scores of 17 or higher, by four-band categorization system (sdqinfo.org).

Those who were excluded based on changing psychopathology class had significantly more clinically significant cases and socioeconomic risks than the included sample but not significantly more than those with persistent psychopathology. This suggests those with persistent and shifting psychopathology have similar symptom severities and socioeconomic backgrounds (eTable 2 in Supplement 1).

**Figure 2.**
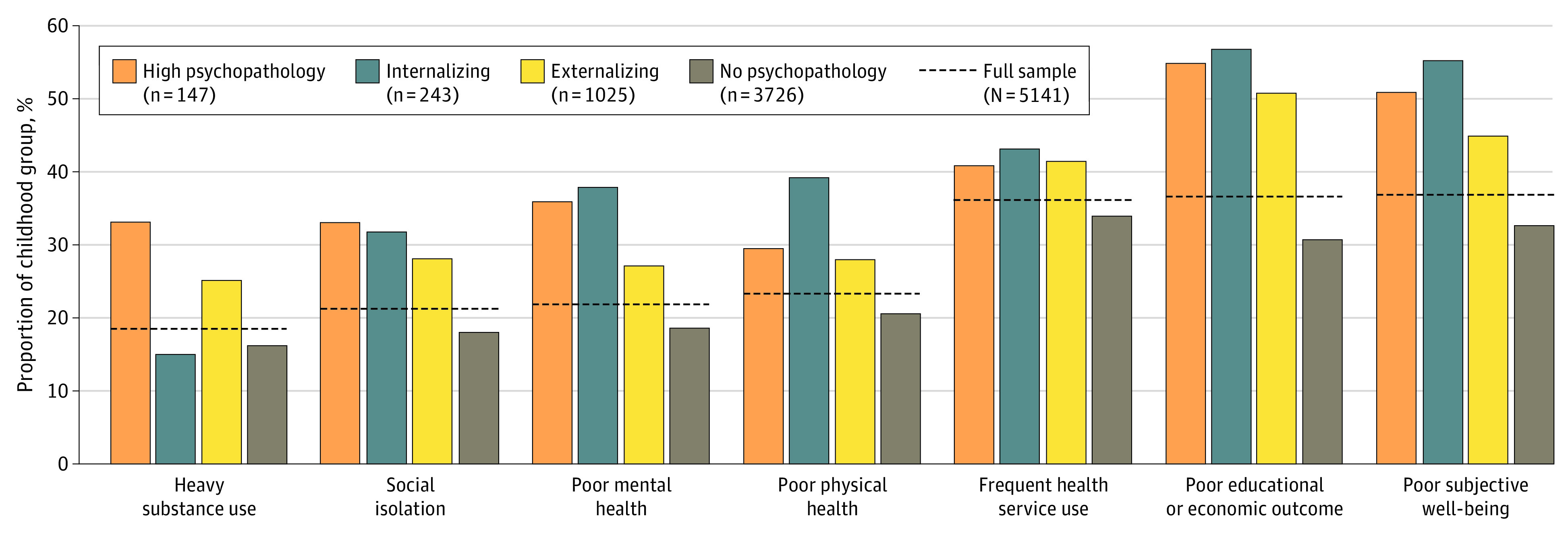
Unadjusted Prevalence of Poor Functional Outcomes for Each Childhood Mental Health Group

Each persistent psychopathology group (internalizing, externalizing, and high psychopathology) had higher crude rates of adverse outcomes in young adulthood than the no psychopathology group. The exception was heavy substance use, which was less common in the internalizing group than the no psychopathology group (Figure 2).

**Figure 3.**
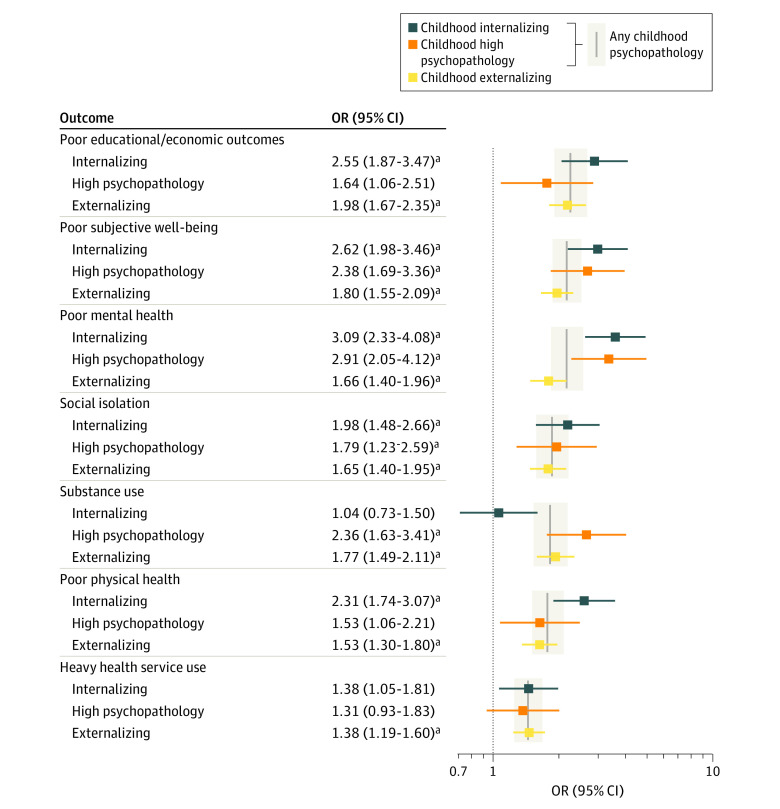
Fully Adjusted Odds of Poor Functional Outcomes for Each Childhood Psychopathology Group and All 3 Combined OR indicates odds ratio. The reference group was no childhood psychopathology. ^a^Significant at a Bonferroni-corrected threshold (*P* < .007).

After controlling for covariates, the odds of all adverse outcomes remained significantly higher for those with any persistent childhood psychopathology compared with the no psychopathology group. Childhood psychopathology groups had an approximately 2-fold increased odds of poor educational/economic outcomes (odds ratio [OR], 2.04; 95% CI, 1.75-2.37), poor mental health (OR, 1.97; 95% CI, 1.70-2.28), and poor subjective well-being (OR, 1.97; 95% CI, 1.72-2.26). Slightly lower but still significant associations were observed with social isolation (OR, 1.72; 95% CI, 1.48-2.00), heavy substance use (OR, 1.69; 95% CI, 1.44-1.98), physical health problems (OR, 1.65; 95% CI, 1.42-1.91), and frequent health service use (OR, 1.37; 95% CI, 1.21-1.57) (Figure 3; eTable 4 in Supplement 1).

There were differences in the patterns of poor outcomes among the childhood psychopathology groups (Figure 3). The internalizing group had the highest odds of several functional outcomes compared with the other psychopathology groups, most notably poor physical health and adverse educational/economic outcomes. Their odds of heavy substance use was significantly lower than the other 2 psychopathology groups and did not significantly differ from the no psychopathology group (Table 2; eTable 5 in Supplement 1). The specific outcome variable most strongly associated with childhood internalizing psychopathology was having few friends at age 20 years (OR, 6.08; 95% CI, 3.13-11.83) (Table 2).

**Table 2.** Adjusted Odds of Adverse Functional Outcomes for Each Childhood Psychopathology Group

Functional outcome	No psychopathology, OR (95% CI) (n = 3726)	High psychopathology (n = 147)		Internalizing (n = 243)		Externalizing (n = 1025)	
		OR (95% CI)^a^	*P* value	OR (95% CI)^a^	*P* value	OR (95% CI)^a^	*P* value
**Poor mental health**							
Any poor mental health	1 [Reference]	2.91 (2.05-4.12)	<.001	3.08 (2.33-4.08)	<.001	1.66 (1.40-1.96)	<.001
Mental health difficulty at age 17 y^b^	1 [Reference]	7.73 (4.53-13.19)	<.001	3.65 (2.16-6.19)	<.001	1.82 (1.23-2.69)	.003
Mental health difficulty at age 20 y	1 [Reference]	3.41 (1.88-6.21)	<.001	4.45 (2.99-6.63)	<.001	1.68 (1.23-2.29)	.001
Saw mental health professional in past y at age 17 y	1 [Reference]	3.51 (2.35-5.27)	<.001	3.46 (2.51-4.77)	<.001	1.64 (1.32-2.03)	<.001
Saw mental health professional in past y at age 20 y	1 [Reference]	1.17 (0.64-2.12)	.62	1.45 (0.98-2.16)	.06	1.42 (1.13-1.77)	.003
**Poor physical health**							
Any poor physical health	1 [Reference]	1.53 (1.06-2.21)	.02	2.31 (1.74-3.07)	<.001	1.53 (1.30-1.80)	<.001
Bad general health at age 17 y^b^	1 [Reference]	1.42 (0.63-3.17)	.40	1.91 (1.04-3.50)	.04	0.74 (0.46-1.19)	.22
Bad general health at age 20 y	1 [Reference]	1.74 (0.82-3.69)	.15	1.69 (0.95-2.98)	.07	1.19 (0.82-1.73)	.36
BMI in obese range at age 17 y^c^	1 [Reference]	3.02 (1.93-4.74)	<.001	2.13 (1.39-3.26)	.001	1.80 (1.40-2.31)	<.001
BMI in obese range at age 20 y^c^	1 [Reference]	1.66 (0.91-3.04)	.10	1.67 (1.10-2.54)	.02	1.60 (1.26-2.04)	<.001
Sleep problems at age 17 y	1 [Reference]	0.80 (0.26-2.44)	.70	1.49 (0.80-2.78)	.21	2.30 (1.68-3.15)	<.001
Sleep problems at age 20 y	1 [Reference]	1.71 (0.81-3.62)	.16	1.72 (1.02-2.91)	.04	1.83 (1.35-2.47)	<.001
**Frequent health service use**							
Any frequent health service use	1 [Reference]	1.31 (0.93-1.83)	.12	1.38 (1.05-1.81)	.02	1.38 (1.19-1.60)	<.001
≥1 Emergency hospital visits per y at age 17 y	1 [Reference]	1.03 (0.66-1.62)	.89	1.07 (0.75-1.55)	.70	1.20 (0.99-1.45)	.06
≥1 Emergency hospital per y at age 20 y	1 [Reference]	1.78 (1.09-2.90)	.02	1.07 (0.72-1.60)	.75	1.50 (1.22-1.84)	<.001
>5 GP visits per y at age 17 y	1 [Reference]	2.10 (1.35-3.27)	.001	2.15 (1.50-3.08)	<.001	1.56 (1.25-1.95)	<.001
>5 GP visits per y at age 20 y	1 [Reference]	0.40 (0.16-1.01)	.05	1.36 (0.89-2.07)	.16	0.94 (0.72-1.23)	.66
**Heavy substance use**							
Any heavy substance use	1 [Reference]	2.36 (1.63-3.41)	<.001	1.04 (0.73-1.50)	.82	1.77 (1.49-2.11)	<.001
Daily smoker at age 17 y	1 [Reference]	2.61 (1.61-4.25)	<.001	1.44 (0.88-2.34)	.15	2.45 (1.93-3.12)	<.001
Daily smoker at age 20 y	1 [Reference]	2.07 (1.25-3.42)	.005	0.77 (0.47-1.25)	.29	1.80 (1.44-2.24)	<.001
Alcohol use disorder at age 17 y	1 [Reference]	4.90 (3.02-7.95)	<.001	0.83 (0.38-1.78)	.63	1.84 (1.37-2.46)	<.001
Alcohol use disorder at age 20 y	1 [Reference]	2.42 (1.28-4.56)	.006	0.80 (0.45-1.41)	.43	1.82 (1.43-2.31)	<.001
**Social isolation**							
Any social isolation	1 [Reference]	1.79 (1.23-2.59)	.002	1.98 (1.48-2.66)	<.001	1.65 (1.40-1.95)	<.001
Few friends at age 17 y	1 [Reference]	2.63 (1.67-4.15)	<.001	2.86 (1.99-4.09)	<.001	1.81 (1.44-2.28)	<.001
Few friends at age 20 y	1 [Reference]	0.99 (0.15-6.53)	.99	6.08 (3.13-11.83)	<.001	3.21 (1.90-5.42)	<.001
No perceived social support at age 17 y	1 [Reference]	0.71 (0.36-1.41)	.33	1.38 (0.90-2.12)	.14	1.37 (1.09-1.72)	.008
No perceived social support at age 20 y	1 [Reference]	2.76 (1.40-5.43)	.003	1.82 (1.04-3.21)	.04	1.74 (1.24-2.44)	.001
**Poor subjective well-being**							
Any poor subjective well-being	1 [Reference]	2.38 (1.69-3.36)	<.001	2.62 (1.98-3.46)	<.001	1.80 (1.55-2.09)	<.001
Low self-esteem at age 17 y	1 [Reference]	1.70 (1.13-2.55)	.01	3.22 (2.41-4.31)	<.001	1.94 (1.63-2.29)	<.001
Low self-esteem at age 20 y	1 [Reference]	1.54 (0.87-2.70)	.14	2.06 (1.43-2.96)	<.001	1.70 (1.37-2.12)	<.001
Dissatisfied with life at age 17 y	1 [Reference]	5.20 (3.52-7.70)	<.001	1.64 (1.07-2.51)	.02	1.86 (1.48-2.35)	<.001
Dissatisfied with life at age 20 y	1 [Reference]	1.65 (1.00-2.72)	.05	2.55 (1.83-3.55)	<.001	1.66 (1.35-2.04)	<.001
**Adverse educational/economic outcomes**							
Any adverse educational/economic outcome	1 [Reference]	1.64 (1.06-2.51)	.03	2.55 (1.87-3.47)	<.001	1.98 (1.67-2.35)	<.001
Low educational attainment at age 17 or 20 y	1 [Reference]	2.79 (1.53-5.10)	.001	2.14 (1.49-3.08)	<.001	2.98 (2.43-3.65)	<.001
NEET at age 20 y	1 [Reference]	5.11 (2.73-9.56)	<.001	5.08 (3.08-8.36)	<.001	3.42 (2.41-4.86)	<.001
Social welfare recipient at age 20 y	1 [Reference]	2.53 (1.57-4.08)	<.001	3.04 (2.17-4.27)	<.001	1.10 (0.86-1.40)	.44
Difficulty making ends meet at age 20 y	1 [Reference]	0.91 (0.40-2.04)	.82	1.50 (0.92-2.44)	.10	1.76 (1.34-2.31)	<.001

Abbreviations: BMI, body mass index (calculated as weight in kilograms divided by height in meters squared); GP, general practitioner; NEET, not in education, employment, or training; OR, odds ratio.

^a^ Adjusted for child’s sex, parent education level, single parenthood, and household income at participant age 13 years.

^b^ Parent-reported outcome.

^c^ Obesity defined as a BMI of 30 or more.

The externalizing group was the only group to have significantly elevated odds of all 7 young adult outcomes, albeit with small ORs relative to the other psychopathology groups. In particular, their odds of poor mental health in adulthood was significantly lower than other psychopathology groups (eTable 5 in Supplement 1) but still significantly higher than the no psychopathology group. The specific outcome most strongly associated with childhood externalizing psychopathology was being NEET at age 20 years (OR, 3.42; 95% CI, 2.41-4.86) (Table 2).

The high psychopathology group had the highest odds of heavy substance use of all psychopathology groups, and their odds of mental health problems and poor subjective well-being were as high as the internalizing group. However, they did not have significantly elevated odds of adverse educational/economic outcomes, poor physical health, or frequent health service use compared with the no psychopathology group, likely due to wide confidence intervals (Figure 3). The specific outcome most strongly associated with being in the high psychopathology group was mental health difficulties at age 17 years (OR, 7.83; 95% CI, 4.53-13.19) (Table 2).

Age-stratified analyses showed that childhood psychopathology remained significantly associated with increased odds of all adverse outcomes, whether they were measured at age 17 years or 20 years. ORs were generally larger for outcomes at age 17 years, with the exception of social isolation (eTable 6 in Supplement 1).

Sex-stratified analyses showed broadly similar results for male and female participants. However, female participants showed stronger associations between childhood psychopathology and both frequent health service use (OR, 1.69; 95% CI, 1.39-2.05) and poor physical health (OR, 1.98; 95% CI, 1.62-2.42). Testing the interaction between childhood psychopathology and sex verified significant moderation by sex (eTables 7 to 9 in Supplement 1).

### Method Triangulation

Using alternative definitions for persistent childhood psychopathology, 123 individuals (5%) had persistently high psychopathology, 433 (17%) had persistent internalizing symptoms, 544 (22%) had persistent externalizing symptoms, and 1420 (56%) had no psychopathology (eAppendix and eTable 10 in Supplement 1). Results using these alternative groups were broadly similar to original results (eTable 12 and eFigure 3 in Supplement 1). The internalizing group had the highest odds of physical health problems of all groups and had a null effect for heavy substance use. The externalizing group had the lowest odds of poor mental health and poor subjective well-being compared with other types of psychopathology (eTable 13 in Supplement 1). Unlike original results, the high psychopathology group had the highest rates of poor mental health and poor subjective well-being of all groups, and the association between internalizing psychopathology and poor educational/economic outcomes was not significant. Results may have varied due to differences in symptom severity or size of groups (eTables 3 and 11 in Supplement 1).

## Discussion

In this general population study of 5141 individuals, those with persisting psychopathology from ages 9 years to 13 years had more difficulties across a range of young adult outcomes compared with those without childhood psychopathology.

### Replicated and Novel Findings

Our findings support 2 well-replicated observations: (1) childhood psychopathology was associated with a range of adverse outcomes in adulthood beyond poor mental health^4,5,34^ and (2) children with externalizing symptoms (with or without comorbid internalizing symptoms) were at significant risk of substance use, unlike those with internalizing symptoms alone.^35,36,37,38^

This study also presents some novel findings. First, poor educational/economic outcomes were as likely as poor mental health for those who experienced persistent childhood psychopathology. Importantly—and replicating other findings^1,4,24^—this finding remained significant after controlling for childhood socioeconomic background. Unlike mental health and well-being, which may fluctuate in and out of pathological ranges throughout the lifespan,^16^ low educational attainment is more likely to have long-lasting effects on the individual’s opportunities.

Second, the childhood externalizing group, while characterized by mostly subthreshold SDQ scores and accounting for 20% of this sample, had the widest range of functional impairments, as evidenced by elevated odds of all 7 young adult outcomes. This mirrors the range of functional outcomes associated with both clinical^9,39,40^ and subclinical^1,37,41^ ADHD symptoms and highlights the need for diverse and personalized supports for these children.

Lastly, the internalizing group had the highest odds of many adverse outcomes, despite a less severe SDQ symptom profile than the high psychopathology group (Figure 1B). It may be that internalizing symptoms, such as depression, anxiety, and interpersonal problems, are particularly harmful to functional development or that these individuals fly under the radar in the absence of disruptive behaviors.^42^ The internalizing group had the highest odds of poor physical health, even when internalizing psychopathology was redefined using SDQ cutoffs. This could be mediated through hypothalamic-pituitary-adrenal axis activation and inflammation associated with internalizing symptoms^43,44,45^; however, unmeasured factors could also be contributing (eg, genetic predisposition, preexisting physical conditions).

### Practical Implications

Targeting individuals with persistent childhood psychopathology for selective preventive interventions may be an efficient way to prevent poor functional outcomes among young adults. The long-term economic benefits of several intervention programs for childhood psychopathology have been estimated to outweigh costs of implementation.^46,47^

More than 50% of all childhood psychopathology groups had adverse educational/economic outcomes by age 20 years (Figure 2), which is not currently acknowledged within child and adolescent mental health services. Improving the integration between schools and mental health services may boost educational attainment in children with psychopathology^48^ and subsequently reduce the risks of employment difficulties.^26^

The odds of frequent health service use were 37% higher in those who experienced persistent childhood psychopathology than those with no psychopathology. While the effect size was small compared with other functional outcomes, the practical importance of this finding is underlined by the high cost of health care. The association appeared to be driven by female participants (as was the association between psychopathology and poor physical health). Further research is needed to test the generalizability of and possible reasons for these sex differences.

Finally, childhood psychopathology was assessed in 2007 to 2011 in this study, but international evidence suggest that youth mental health has worsened since then.^7,49^ Given that the prevalence of childhood psychopathology may have increased, the scale of effects on young adulthood functioning may also have grown.^50^

### Limitations

This study has limitations. First, the sample may have been biased by the exclusion of children without SDQ data from 3 waves (although sampling weights were used to mitigate this) and those who changed classes from ages 9 to 13 years. We excluded this latter group because acknowledging all possible transitions (eg, internalizing to externalizing) would have resulted in many qualitatively different groups. Participants who changed classes showed high levels of socioeconomic and clinical risk (eTable 2 in Supplement 1) and should be studied in future. Second, SDQs were reported by parents, while outcomes were predominantly reported by participants. Both sources may be biased, but having different informants for exposure and outcome reduces the risk of common method variance. Third, unmeasured confounding may exist (eg, early-life adversity or trauma). Fourth, varying levels of missing data across outcomes may limit direct comparisons between them (eFigure 1 in Supplement 1). Fifth, certain exposures and outcomes overlapped conceptually (eg, SDQ peer problems and social isolation), which may change the interpretation of results to continuation rather than diversification of problems.

## Conclusions

In this cohort study of 5141 individuals, we found trajectories of persistent childhood psychopathology were associated with widespread functional impairments in late adolescence and early adulthood. Findings point to the need for improved public screening and treatment of child psychopathology.

## Notes

Supplement 1. **eMethods 1.** Measure Details **eMethods 2.** Sampling Details **eTable 1.** Definitions of Each Outcome Variable and Descriptive Statistics for the Sample **eFigure 1.** Flowchart of Participation in the GUI Longitudinal Study and Exclusions Leading to the Final Sample Size **eTable 2.** Differences in Demographic and Clinical Characteristics Between the Included Sample and Participants Who Were Excluded Based on a Changing Childhood Psychopathology Group Between Ages 9 and 13 Years **eFigure 2.** Cutoffs for Dichotomised Variables **eTable 3.** Mean SDQ Scores for the Full Sample (Grey Rows) and Each Childhood Psychopathology Group at Age 9 Years (Top) and Age 13 Years (Bottom) **eTable 4.** Odds of Poor Functional Outcomes in Those With Any Persisting Psychopathology (ie, High, Externalising or Internalising), Unadjusted and Adjusted for Potential Confounds **eTable 5.** Odds Ratios of Adverse Adult Outcomes Between the Childhood Psychopathology Groups Fully Adjusted for Socioeconomic Factors and Sex **eTable 6.** Results of Age-Stratified Analyses **eTable 7.** Results of Sex-Stratified Analyses (Any Psychopathology) **eTable 8.** Results of Sex-Stratified Analyses (Externalising Psychopathology) **eTable 9.** Results of Sex-Stratified Analyses (Internalising Psychopathology) **eAppendix.** Results of Method Triangulation **eTable 10.** Definitions and Group Sizes for the Alternative Childhood Psychopathology Groups Used in the Sensitivity Analysis **eTable 11.** Mean SDQ Scores for the Alternative Persistent Psychopathology Groups Used in the Sensitivity Analysis (n = 2520) **eTable 12.** Odds of Adverse Adult Outcomes in Those With Any Persistent Psychopathology From Age 9 to 13 Years Using Alternative Group Definitions for the Sensitivity Analysis **eTable 13.** Odds Ratios (OR) of Difference Between Childhood Groups in the Odds of Each Adverse Outcome in Young Adulthood (Reference Group: Low Psychopathology) **eFigure 3.** Adjusted Odds of Poor Young Adult Outcomes for Childhood Mental Health Groups Used in the Sensitivity Analysis
eReferences.

Supplement 2.
Data Sharing Statement
