# Supplementary material for: Functional Outcomes Among Young People With Trajectories of Persistent Childhood Psychopathology
Source: JAMA Netw Open. 2023 Sep 29;6(9):e2336520. doi: 10.1001/jamanetworkopen.2023.36520 (PMC10543080; doi:10.1001/jamanetworkopen.2023.36520)
Supplement: Supplement 1. — eMethods 1. Measure Details eMethods 2. Sampling Details eTable 1. Definitions of Each Outcome Variable and Descriptive Statistics for the Sample eFigure 1. Flowchart of Participation in the GUI Longitudinal Study and Exclusions Leading to the Final Sample Size eTable 2. Differences in Demographic and Clinical Characteristics Between the Included Sample and Participants Who Were Excluded Based on a Changing Childhood Psychopathology Group Between Ages 9 and 13 Years eFigure 2. Cutoffs for Dichotomised Variables eTable 3. Mean SDQ Scores for the Full Sample (Grey Rows) and Each Childhood Psychopathology Group at Age 9 Years (Top) and Age 13 Years (Bottom) eTable 4. Odds of Poor Functional Outcomes in Those With Any Persisting Psychopathology (ie, High, Externalising or Internalising), Unadjusted and Adjusted for Potential Confounds eTable 5. Odds Ratios of Adverse Adult Outcomes Between the Childhood Psychopathology Groups Fully Adjusted for Socioeconomic Factors and Sex eTable 6. Results of Age-Stratified Analyses eTable 7. Results of Sex-Stratified Analyses (Any Psychopathology) eTable 8. Results of Sex-Stratified Analyses (Externalising Psychopathology) eTable 9. Results of Sex-Stratified Analyses (Internalising Psychopathology) eAppendix. Results of Method Triangulation eTable 10. Definitions and Group Sizes for the Alternative Childhood Psychopathology Groups Used in the Sensitivity Analysis eTable 11. Mean SDQ Scores for the Alternative Persistent Psychopathology Groups Used in the Sensitivity Analysis (n = 2520) eTable 12. Odds of Adverse Adult Outcomes in Those With Any Persistent Psychopathology From Age 9 to 13 Years Using Alternative Group Definitions for the Sensitivity Analysis eTable 13. Odds Ratios (OR) of Difference Between Childhood Groups in the Odds of Each Adverse Outcome in Young Adulthood (Reference Group: Low Psychopathology) eFigure 3. Adjusted Odds of Poor Young Adult Outcomes for Childhood Mental Health Groups Used in the Sensitivity Analysi [file jamanetwopen-e2336520-s001.pdf]

Supplemental Online Content

Dooley N, Kennelly B, Arseneault L, et al. Functional outcomes among young people with trajectories of persistent childhood psychopathology. *JAMA Netw Open*. 2023;6(9):e2336520. doi:10.1001/jamanetworkopen.2023.36520

eMethods 1. Measure Details

eMethods 2. Sampling Details

eTable 1. Definitions of Each Outcome Variable and Descriptive Statistics for the Sample

eFigure 1. Flowchart of Participation in the GUI Longitudinal Study and Exclusions Leading to the Final Sample Size

eTable 2. Differences in Demographic and Clinical Characteristics Between the Included Sample and Participants Who Were Excluded Based on a Changing Childhood Psychopathology Group Between Ages 9 and 13 Years

eFigure 2. Cutoffs for Dichotomised Variables

eTable 3. Mean SDQ Scores for the Full Sample (Grey Rows) and Each Childhood Psychopathology Group at Age 9 Years (Top) and Age 13 Years (Bottom)

eTable 4. Odds of Poor Functional Outcomes in Those With Any Persisting Psychopathology (ie, High, Externalising or Internalising), Unadjusted and Adjusted for Potential Confounds

eTable 5. Odds Ratios of Adverse Adult Outcomes Between the Childhood Psychopathology Groups Fully Adjusted for Socioeconomic Factors and Sex

eTable 6. Results of Age-Stratified Analyses

eTable 7. Results of Sex-Stratified Analyses (Any Psychopathology)

eTable 8. Results of Sex-Stratified Analyses (Externalising Psychopathology)

eTable 9. Results of Sex-Stratified Analyses (Internalising Psychopathology)

eAppendix. Results of Method Triangulation

eTable 10. Definitions and Group Sizes for the alternative Childhood Psychopathology Groups Used in the Sensitivity Analysis

eTable 11. Mean SDQ Scores for the Alternative Persistent Psychopathology Groups Used in the Sensitivity Analysis (n = 2520)

eTable 12. Odds of Adverse Adult Outcomes in Those With Any Persistent Psychopathology From Age 9 to 13 Years Using Alternative Group Definitions for the Sensitivity Analysis

eTable 13. Odds Ratios (OR) of Difference Between Childhood Groups in the Odds of Each Adverse Outcome in Young Adulthood (Reference Group: Low Psychopathology)

eFigure 3. Adjusted Odds of Poor Young Adult Outcomes for Childhood Mental Health Groups Used in the Sensitivity Analysis

© 2023 Dooley N et al. *JAMA Network Open*.

eReferences.

This supplementary material has been provided by the authors to give readers additional information about their work.

© 2023 Dooley N et al. *JAMA Network Open*.

eMethods 1. Measure Details

Distributions of continuous variables with cut-points indicated are shown in eFigure 2. Justification for cut-points are provided below.

AUDIT (15+)
The Alcohol Use Disorders Identification Test (AUDIT) has been developed from a six-country WHO collaborative project as a screening instrument for hazardous and harmful alcohol consumption. Responses to each question are scored from 0 to 4, giving a maximum possible score of 40. Based on empirical research, the AUDIT manual suggests scores from 8 to 14 suggest hazardous or harmful alcohol consumption, and scores of over 15 indicates the likelihood of alcohol dependence or "moderate-severe alcohol use disorder"[1].

Number of friends (<3)
We used the lowest available level for number of friends in the dataset, which was 0-2. This category was likely created due to small group sizes for specific numerical levels, and to avoid identification of specific participants. Our cut-off is supported by Brugha et al.[2], who found that primary social group of 3 or less predicted poorer mental health at follow-up.

Rosenberg Self-esteem scale (<10 / <9)
A six-item version of the Rosenberg self-esteem scale was administered at age 17 and 20. Each item was rated on a four-point scale (strongly disagree, disagree, agree & strongly agree; numeric weights = 0, 1, 2 & 3) providing total scores ranging from 0 to 18, with higher scores indicative of higher self-esteem. Items included (1) on the whole, I am satisfied with myself; (2) at times, I think I am no good at all; (3) I am able to do things as well as most other people; (4) I certainly feel useless at times; (5) All in all, I am inclined to feel that I am a failure; (6) I take a positive attitude towards myself. Due to the lack of consensus on cut-offs for this scale, we chose the bottom quartile of scores to reflect "low" self-esteem for each age-group (<10 at age 17, or <9 at age 20).

Satisfaction with life (<5)
This one-item variable was not a validated measure. The question was: "*if you were to describe how satisfied you are with your own life in general how would you rate it on a scale of 0 to 10, 0 meaning you are extremely unsatisfied with your life in general, and 10 meaning that you are extremely satisfied with your life.*" As indicated, responses were provided on a 11-point scale from 0 "extremely unsatisfied" to 10 "extremely satisfied". Due to the lack of guiding cut-offs or literature on this measure, we chose anything below the midpoint (5) to indicate dis-satisfaction with life.

Body Mass Index (30+)
Weight was measured in-person by GUI researchers, to the nearest 0.5 kg using a medically approved flat mechanical scales. Participants were advised to wear light clothing. Height was measured to the nearest mm using a height measuring stick. For generalisability to other adult studies, and given the similarity between adult cut-offs and recommended cut-offs for obesity in 17-

© 2023 Dooley N et al. *JAMA Network Open*.

year-olds (29.5-29.9), we chose to apply the standard adult cut-off for obesity, as defined by the World Health Organisation (≥ 30).

GP visits (>5)

Participants were asked *"In the last 12 months, how many times have you seen or consulted, or talked on the phone with <a general practitioner> about your physical, emotional or mental health?"*. Response options were provided on the numeric-categorical scales shown in Figure S1. The cut-point of >5 GP visits (i.e. 6 or more) in the past year was based on findings from a UK survey of over 4,000 individuals[3]. It found that the average number of GP visits per year was 5.5 for the whole sample but between 7 and 12 for those with severe mental health symptoms.

Leaving Certificate examinations (<300)

The leaving certificate is the final exam of the Irish secondary school system and the university matriculation examination in Ireland. In the established leaving certificate, students sit exams for a minimum of 6 subjects with ~100 points available for each subject. Students therefore typically score between 0 and 600 points. We chose 300 as the cut-off for low educational attainment as it corresponded roughly to the lowest quintile. A GUI report showed that less than half of those with points in the lowest quintile went onto higher education (42%), post-leaving certificate course (37%), or other further education (18%)[4].

A small number of participants reported their leaving certificate results at age 17 (n=371), with the vast majority reporting at age 20. These responses were merged into one variable, with the most recent result taken if two responses were provided.

Equivalised income

One socioeconomic variable which was included as a covariate in adjusted analyses was household equivalised income (from age 13 data collection). An equivalence scale was used to assign a "weight" to each household member (weight of 1 to the first adult in the household, 0.66 to each subsequent adult aged 14+ and 0.33 to each child <14 years). The sum of these weights in each household gives the household's equivalised size – the size of the household in adult equivalents. Disposable household income was recorded as total gross household income less statutory deductions of income tax and social insurance contributions. Household equivalised income was the disposable household income divided by equivalised household size.

Parent Education

The education level of primary and secondary caregivers were merged according to the dominance criterion. That is, when valid responses were provided by both parents, the higher level of education was taken; when only one parent had responded, that was taken as the highest level of education. Levels included: none/primary school, lower secondary (e.g. Junior Cert), higher secondary (e.g. Leaving Cert) or technical vocational, non-degree certificate, primary degree, and postgraduate degree.

© 2023 Dooley N et al. *JAMA Network Open*.

eMethods 2. Sampling Details

A two-stage recruitment process of the cohort was conducted.[5] First, 910 national schools were randomly selected for involvement, as the primary sampling units. Within these schools, children aged 9 and their families were invited to partake. Second, participants were selected to reflect the distribution of social and economic factors across the Irish population of nine-year-olds at that time.

Additional sample weights were created by comparing the sample with the children aged 9 in the 2006 Irish Census, based on several key socio-demographic variables and readjusted for over/under representation. Similarly, the sample was also re-weighted for attrition between the waves[6]. Thus, the statistics reported in this study are representative of 9-year-olds who were residing in Ireland, who continued to live in Ireland at 17-20 years of age (target population), and who exhibited similar mental health presentations at ages 9 and 13.

Cohort follow-ups took places when participants were approximately 13 years (n= 7,423; 88% retention), 17 years (n= 6,216; 74% retention from baseline), and 20 years (n=5,190, 61% retention from baseline). In the 17-year-old data, most participants (81.5%) were aged 17, however almost a fifth were aged 18 (18.5%) and a few were aged 16 (<1%). Similarly, in the final wave while the most common age was 20 years (90.8%), some participants were aged 19 (1.0%) and 21 (8.1%). Age was only provided in these categorical terms in the openly accessible GUI datasets.

The data of births of the cohort ranged from 1 Nov 1997 to 31 Oct 1998. Data collection occurred in 2007-2008 (age 9 data); 2011-2012 (age 13 data); 2015-2016 (age 17 data); and 2018-2019 (age 20 data).

© 2023 Dooley N et al. *JAMA Network Open*.

eTable 1. Definitions of Each Outcome Variable and Descriptive Statistics for the Sample

Max sample size at age 17 and 20 = 5,141 and 4,024.

Young adult outcome:	% of sample [b]	Definition
Mental Health Problem	21.9% (n=1,123)	Any of the following…
Mental health problem @17 [a]	3.4% (n=171)	"Yes" to "Does [young person] have mental health difficulty?" (Parent-reported)
Mental health problem @20	7.3% (n=294)	"Yes" to "Do you have any on-going chronic physical or mental health problem, illness or disability?" *AND* problem subsequently coded as mental illness by GUI data analysts
Saw mental health professional @17	11.6% (n=596)	1 or more to "In the last 12 months, how many times have you seen or consulted, or talked on the phone with [other professional, psychologist, psychiatrist, counsellor, etc] about your physical, emotional or mental health?"
Saw mental health professional @20	14.6% (n=588)	1 or more to "In the last 12 months, how many times have you seen or consulted, or talked on the phone with a [psychologist, psychiatrist, counsellor, etc] about your physical, emotional or mental health?"
Physical Health Problem	23.3% (n=1,198)	Any of the following…
Obesity @17	7.4% (n=368)	Body Mass Index (BMI) 30 or over
Obesity @20	12.7% (n=494)	Body Mass Index (BMI)30 or over
Sleep problems @17	4.3% (n=219)	"Yes, a lot of difficulty", to "Do you have any difficulty with sleep?"
Sleep problems @20	6.6% (n=267)	"Yes, a lot of difficulty", to "Do you have any difficulty with sleep?"
Bad general health @17 [a]	2.8% (n=142)	"Almost always unwell" or "Sometimes quite ill", to "In general, how would you describe [young person's] health in the past year?"
Bad general health @20	4.9% (n=198)	"Fair" or "poor", to "In general, how would you say your current health is?"
Heavy substance use	18.5% (n=951)	Any of the following…
Daily cigarette smoker @17	7.4% (n=374)	"Daily" to "Do you currently smoke daily, occasionally or not at all?"
Daily cigarette smoker @20	13.9% (n=555)	"Smoke daily" to "Which of the following best describes you: Smoke daily; Only ever tried once or twice; Used to but not now; Occasionally"
Excessive alcohol-use @17	6.3% (n=270)	AUDIT questionnaire total score 15 or more
Excessive alcohol-use @20	13.8% (n=520)	AUDIT questionnaire total score 15 or more
Frequent health service use	36.1% (n=1,858)	Any of the following…
≥ 1 A&E visits in past year @17	15.9% (n=815)	1 or more to "In the last 12 months, how many times have you seen or consulted, or talked on the phone with [A&E] about your physical, emotional or mental health?"
≥ 1 A&E visits in past year @20	17.7% (n=711)	1 or more to "In the last 12 months, how many times have you seen or consulted, or talked on the phone with [A&E] about your physical, emotional or mental health?"
>5 GP visits per year @17	10.9% (n=554)	Over 5 to "In the last 12 months, how many times have you seen or consulted, or talked on the phone with [GP] about your physical, emotional or mental health? "
>5 GP visits per year @20	11.8% (n=473)	Over 5 to "In the last 12 months, how many times have you seen or consulted, or talked on the phone with [GP] about your physical, emotional or mental health? "
Social Isolation	21.2% (n=1,085)	Any of the following…
Few friends @17	9.2% (n=467)	<3 to "How many friends do you normally hang around with?"
Few friends @20	2.3% (n=94)	<3 to "How many friends do you have?"
No social support @17	9.7% (n=494)	"No" to "Is there an adult in your life you can usually turn to for help and advice?"

© 2023 Dooley N et al. *JAMA Network Open*.

**Young adult outcome:**	**% of sample [b]**	**Definition**
No social support @20	5.3% (n=212)	"No" to "Is there someone in your life you can usually turn to for help and advice?"
Educational/economic outcomes	36.6% (n=1,483)	Any of the following…
Low educational attainment	23.0% (n=835)	"300 or less" to "How many points did you get in total in the Leaving Certificate examinations?" (Secondary school finals in Ireland)
NEET @20	4.8% (n=193)	"Unemployed / unable to work or study due to disability or illness / minding family or home / taking a year out or travelling / taking the summer off" to "Please indicate what your main status is with regard to work or other activity. Indicate the category which best applies (this month)."
Social welfare recipient @20	14.0% (n=559)	"5% or more" to "what proportion of your total income would you say comes from social welfare payments of any kind" (reference: 0%)
Difficulty making ends meet @ 20	8.5% (n=339)	"With great difficulty" or "With difficulty" to "Concerning your total monthly or weekly income, with which degree of ease or difficulty are you able to make ends meet?" (Reference: "with some difficulty / fairly easily / easily / very easily")
Poor subjective wellbeing	36.9% (n=1,883)	Any of the following…
Low self-esteem @17	23.1% (n=1,167)	Bottom quartile of scores in Rosenberg self-esteem scale
Low self-esteem @20	15.5% (n=620)	Bottom quartile of scores in Rosenberg self-esteem scale
Dissatisfied with life @17	8.6% (n=439)	<5 to "If you were to describe how satisfied you are with your own life in general, how would you rate it on a scale of 0 to 10 (where 0 = extremely unsatisfied; 10 = extremely satisfied."
Dissatisfied with life @20	18.5% (n=739)	<5 to "If you were to describe how satisfied you are with your own life in general, how would you rate it on a scale of 0 to 10 (where 0 = extremely unsatisfied; 10 = extremely satisfied."

a. parent-reported
b. % of sample with valid/non-missing response to question
NEET = Not in Education, Employment or Training

© 2023 Dooley N et al. *JAMA Network Open*.

eFigure 1. Flowchart of Participation in the GUI Longitudinal Study and Exclusions Leading to the Final Sample Size

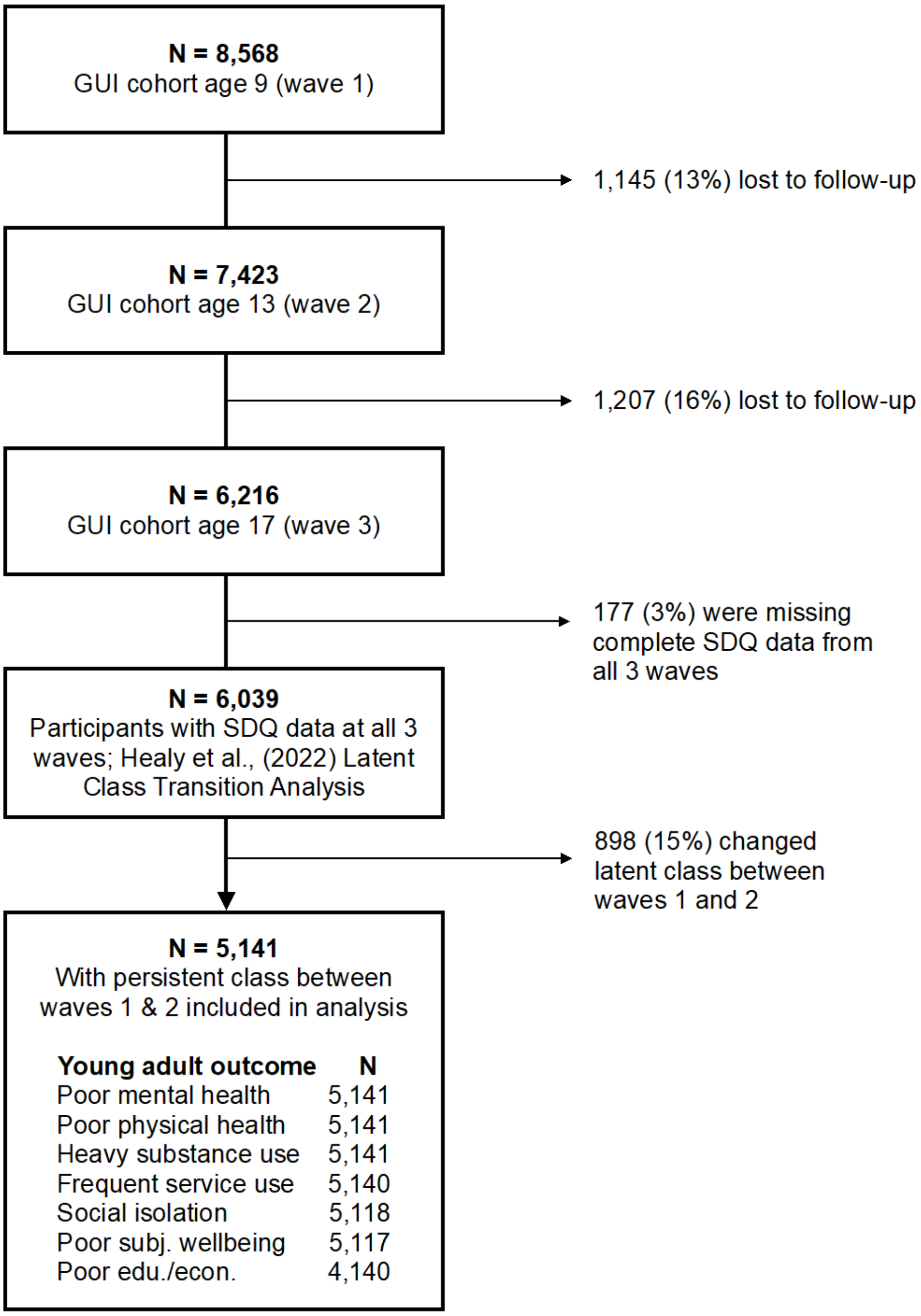

© 2023 Dooley N et al. *JAMA Network Open*.

eTable 2. Differences in Demographic and Clinical Characteristics Between the Included Sample and Participants Who Were Excluded Based on a Changing Childhood Psychopathology Group Between Ages 9 and 13 Years

**Characteristics**	(i) **Full included sample** (same class aged 9-13)	(ii) **Included sub-sample** (Persistent psychopathology; aged 9-13)	(iii) **Excluded** (changed class between ages 9 and 3)	Group difference [c] (i — ii)	Group difference [c] (ii — iii)
Age 9 high SDQ total problem score (%)[a]	236 (4.7%)	236 (17.1%)	180 (17.8%)	221.8***	0.14
Age 13 high SDQ total problem score (%)[a]	206 (4.1%)	206 (14.9%)	176 (17.2%)	243.4***	2.11
Male sex (%)	2553 (50.9%)	760 (55.1%)	529 (51.6%)	0.13	2.73
Low parental education (%)[b]	644 (12.9%)	250 (18.1%)	221 (21.5%)	51.3***	4.07*
Single parent home (%)	854 (17.0%)	353 (25.6%)	261 (25.5%)	30.7***	<0.01
Mean income decile (SD)	6.21 (2.86)	5.56 (2.78)	5.77 (2.85)	4.14***	-1.57

All statistics are weighted to account for sociodemographic sampling biases and attrition. Weighted Ns using age 17 weights were: (i) 5,014 (ii) 1,380 and (iii) 1025. Unweighted Ns were: (i) 5,141 (ii) 1,178 and (iii) 898.
Parental education, income and single parenthood recorded at participant age 13.
a. High SDQ scores defined as scores of 17 or higher (sdqinfo.org)
b. Low parental education defined as homes where parent(s) did not complete Leaving Certificate (final secondary school examinations) or an equivalent
c. Group difference values are Chi-square statistics or t-values (T-tests) for frequencies and means respectively (*p < .05, **p < .01, *** p < .001)

© 2023 Dooley N et al. *JAMA Network Open*.

eFigure 2. Cutoffs for Dichotomised Variables

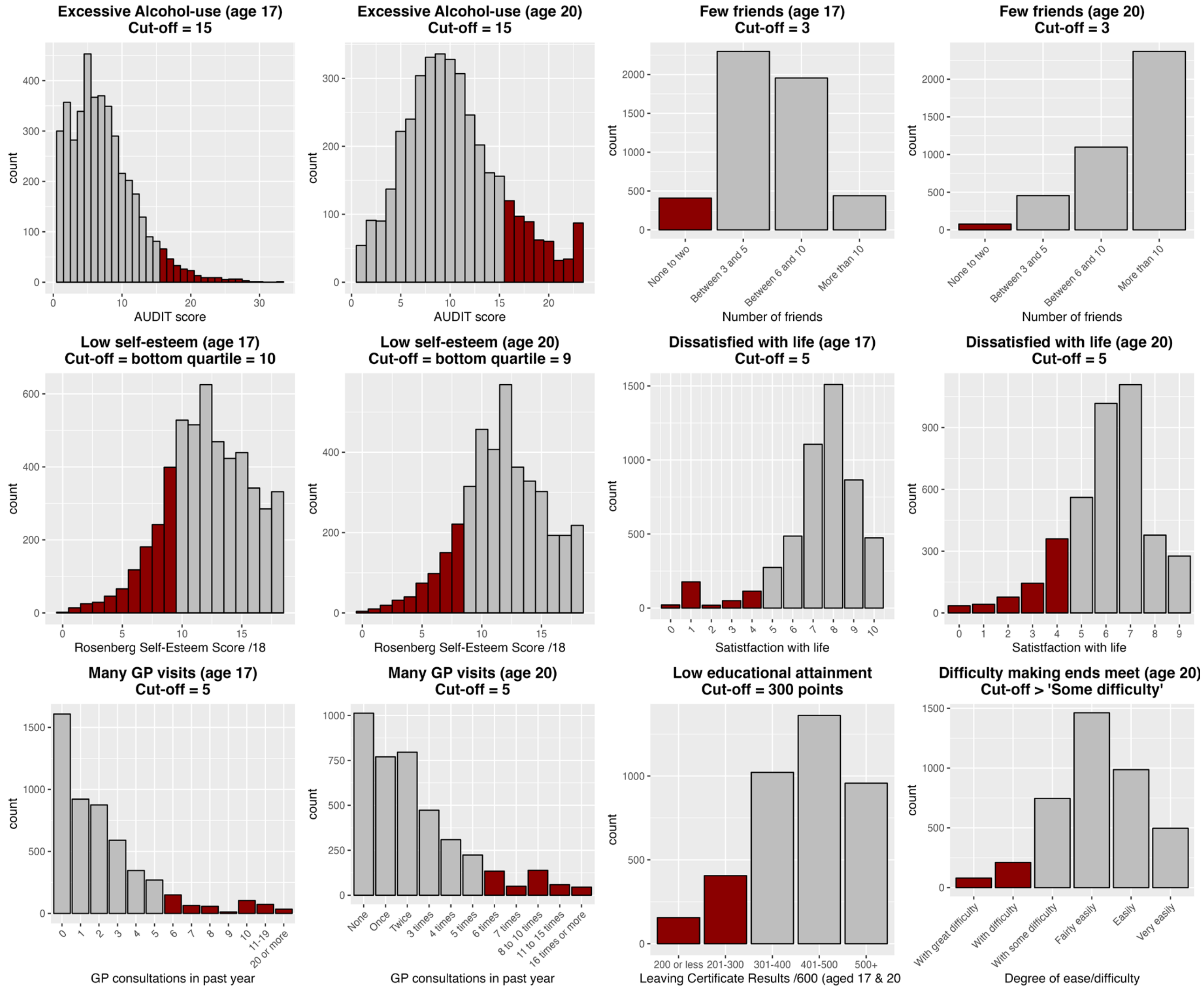

© 2023 Dooley N et al. *JAMA Network Open*.

eTable 3. Mean SDQ Scores for the Full Sample (Grey Rows) and Each Childhood Psychopathology Group at Age 9 Years (Top) and Age 13 Years (Bottom)

		Mean (SD)			
	N	Emotional	Peer	Hyperactivity/ Inattention	Conduct
**Age 9**	5,141	1.81 (1.83)	0.91 (1.21)	2.75 (2.34)	1.10 (1.31)
Low Psychopathology	3,726	1.46 (1.54)	0.64 (0.82)	1.98 (1.70)	0.72 (0.91)
Externalising	1,025	2.53 (1.97)	1.00 (0.95)	5.70 (2.06)	2.44 (1.42)
Internalising	243	3.80 (2.25)	3.66 (1.23)	3.23 (2.12)	1.21 (1.12)
High Psychopathology	147	5.22 (2.50)	5.00 (1.82)	7.09 (2.25)	4.57 (1.91)
**Age 13**	5,141	1.56 (1.77)	0.90 (1.28)	2.28 (2.22)	0.91 (1.20)
Low Psychopathology	3,726	1.17 (1.40)	0.64 (0.89)	1.51 (1.45)	0.59 (0.83)
Externalising	1,025	2.37 (1.91)	0.95 (1.01)	5.20 (1.99)	2.02 (1.30)
Internalising	243	3.94 (2.23)	4.21 (1.54)	2.72 (2.16)	1.00 (1.06)
High Psychopathology	147	5.10 (2.45)	3.94 (1.80)	7.30 (2.29)	4.67 (1.78)

© 2023 Dooley N et al. *JAMA Network Open*.

eTable 4. Odds of Poor Functional Outcomes in Those With Any Persisting Psychopathology (ie, High, Externalising or Internalising), Unadjusted and Adjusted for Potential Confounds

	Unadjusted			Fully Adjusted ±		
	OR (95% CI)	z	p	OR (95% CI)	z	p
Mental health problems	**1.87 (1.63-2.15)**	8.83	<.001	**1.97 (1.70-2.28)**	8.95	<.001
Physical health problems	**1.66 (1.45-1.91)**	7.27	<.001	**1.65 (1.42-1.91)**	6.62	<.001
Substance use	**1.66 (1.43-1.92)**	6.66	<.001	**1.69 (1.44-1.98)**	6.48	<.001
Frequent health service use	**1.39 (1.23-1.57)**	5.22	<.001	**1.37 (1.21-1.57)**	4.72	<.001
Social isolation	**1.88 (1.64-2.27)**	8.81	<.001	**1.72 (1.48-2.00)**	7.04	<.001
Poor educational/economic outcomes	**2.47 (2.15-2.85)**	12.53	<.001	**2.04 (1.75-2.37)**	9.13	<.001
Poor subjective wellbeing	**1.86 (1.64-2.10)**	9.79	<.001	**1.97 (1.72-2.26)**	9.88	<.001

Bold effects refer to those significant at Bonferoni-corrected threshold (p < .007)

± Adjusted for child's sex, parent education level, single-parenthood & household income at age 13

© 2023 Dooley N et al. *JAMA Network Open*.

eTable 5. Odds Ratios of Adverse Adult Outcomes Between the Childhood Psychopathology Groups Fully Adjusted for Socioeconomic Factors and Sex

	**High** Vs **Externalising-only**					**High** Vs **Internalising-only**					**Internalising** Vs **Externalising**				
	**OR**	**Lower**	**Upper**	**z**	**p**	**OR**	**Lower**	**Upper**	**z**	**p**	**OR**	**Lower**	**Upper**	**z**	**p**
Poor Mental Health	**1.83**	**1.26**	**2.65**	**3.19**	**.001**	0.92	0.58	1.44	-0.38	.70	**1.90**	**1.41**	**2.57**	**4.19**	**<.001**
Poor Physical Health	1.11	0.75	1.63	0.50	.62	0.60	0.36	0.98	-2.02	.04	1.49	1.09	2.03	2.51	.01
Heavy substance use	1.29	0.88	1.90	1.31	.19	**2.31**	**1.37**	**3.91**	**3.12**	**.002**	0.60	0.41	0.88	2.66	.008
Frequent health service use	0.93	0.65	1.33	-0.38	.70	0.93	0.59	1.47	-0.31	.76	1.00	0.75	1.35	0.01	.99
Social Isolation	1.07	0.73	1.59	0.36	.72	0.89	0.55	1.46	-0.44	.66	1.19	0.87	1.63	1.09	.28
Poor subjective wellbeing	1.34	0.93	1.94	1.58	.12	0.87	0.56	1.35	-0.62	.53	1.44	1.07	1.94	2.42	.02
Educational/economic outcomes	0.93	0.59	1.45	-0.33	0.74	0.65	0.38	1.13	-1.51	0.13	1.26	0.90	1.75	1.35	0.18

Bold effects refer to those significant at Bonferoni-corrected threshold (p < .007)

© 2023 Dooley N et al. *JAMA Network Open*.

eTable 6. Results of Age-Stratified Analyses

Fully adjusted effect of any childhood psychopathology on each functional outcome, split by age of outcome measurement.

	OR (95% CI)	z	p
Poor mental health (age 17 or 20)	1.97 (1.70-2.28)	8.95	<.001
Age 17	2.18 (1.81-2.62)	8.29	<.001
Age 20	1.77 (1.47-2.14)	5.99	<.001
Poor physical health (age 17 or 20)	1.65 (1.42-1.91)	6.62	<.001
Age 17	1.96 (1.64-2.34)	7.34	<.001
Age 20	1.59 (1.33-1.90)	5.10	<.001
Heavy substance use (age 17 or 20)	1.69 (1.44-1.98)	6.48	<.001
Age 17	2.04 (1.68-2.48)	7.24	<.001
Age 20	1.51 (1.22-1.89)	3.69	<.001
Frequent health service use (age 17 or 20)	1.37 (1.21-1.57)	4.72	<.001
Age 17	1.42 (1.22-1.66)	4.59	<.001
Age 20	1.30 (1.10-1.54)	3.10	0.002
Social isolation (age 17 or 20)	1.72 (1.48-2.00)	7.04	<.001
Age 17	1.72 (1.46-2.02)	6.48	<.001
Age 20	2.15 (1.65-2.81)	5.68	<.001
Poor educational/economic outcome (age 20)	2.04 (1.75-2.37)	9.13	<.001
Age 17	—	—	—
Age 20	2.04 (1.75-2.37)	9.13	<.001
Poor subjective wellbeing (age 17 or 20)	1.97 (1.72-2.26)	9.88	<.001
Age 17	2.06 (1.78-2.38)	9.65	<.001
Age 20	1.78 (1.51-2.11)	6.73	<.001

Odds ratios refer to the group difference between those with childhood psychopathology (internalising/externalising/high psych.) and those with no childhood psychopathology, adjusted for sex and socioeconomic factors.

© 2023 Dooley N et al. *JAMA Network Open*.

eTable 7. Results of Sex-Stratified Analyses

Fully adjusted effect of any childhood psychopathology on each functional outcome for males and females separately.

	Females			Males			
	608 persistent psychopathology Vs 1697 no persistent psych. [a]			767 persistent psychopathology Vs 1630 no persistent psych. [a]			
**Outcome (age 17/20)**	**OR (95% CI)**	**z**	**p**	**OR (95% CI)**	**z**	**p**	**Interaction Effect** [b]
Poor mental health	1.81 (1.47-2.24)	5.52	<.001	2.13 (1.73-2.63)	7.09	<.001	z=-1.70, p=.09
Poor physical health	1.98 (1.62-2.42)	6.69	<.001	1.37 (1.10-1.70)	2.80	0.005	**z=3.09, p=.002**
Heavy substance use	1.83 (1.44-2.32)	4.96	<.001	1.61 (1.30-1.99)	4.39	<.001	z=0.83, p=.41
Frequent health service use	1.69 (1.39-2.05)	5.34	<.001	1.13 (0.94-1.35)	1.27	0.204	**z=2.96, p=.003**
Social isolation	1.86 (1.48-2.32)	5.43	<.001	1.67 (1.36-2.05)	4.90	<.001	z=1.41, p=.16
Poor subjective wellbeing	1.96 (1.62-2.38)	6.86	<.001	1.99 (1.65-2.41)	7.20	<.001	z=0.19, p=.85
Poor educational/ economic outcome	2.12 (1.69-2.65)	6.55	<.001	2.06 (1.67-2.55)	6.70	<.001	z=0.25, p=.80

Odds ratios refer to the group difference between those with persistent childhood psychopathology (internalising/externalising/high psych.) and those with no childhood psychopathology, adjusted for sex and socioeconomic factors.
a. Group sizes refers to those included in fully-adjusted model (i.e., with data on all covariates); Lower N available for educational/economic outcomes analysis (459 females & 576 males with any persistent psychopathology)
b. Interaction effect refers to multiplicative interaction between childhood psychopathology and sex, adjusted for main effects of childhood psychopathology, sex and socioeconomic factors.

© 2023 Dooley N et al. *JAMA Network Open*.

eTable 8. Results of Sex-Stratified Analyses (Externalising Psychopathology)

Fully adjusted effect of childhood externalising on each functional outcome for males and females separately.

	Females			Males		
	443 persistent externalising Vs 1697 no persistent psych.[a]			553 persistent externalising Vs 1630 no persistent psych.[a]		
**Outcome (age 17/20)**	**OR**	**z**	**p**	**OR**	**z**	**p**
Poor mental health	1.77 (1.40-2.24)	4.72	<.001	1.57 (1.23-1.99)	3.64	<.001
Poor subjective wellbeing	1.94 (1.57-2.41)	6.02	<.001	1.68 (1.36-2.08)	4.85	<.001
Substance use	1.78 (1.36-2.32)	4.25	<.001	1.78 (1.41-2.24)	4.90	<.001
Poor educational/economic outcome	2.57 (1.99-3.31)	7.29	<.001	1.67 (1.32-2.11)	4.25	<.001
Poor physical health	1.72 (1.37-2.16)	4.70	<.001	1.34 (1.05-1.71)	2.35	0.02
Frequent health service use	1.63 (1.31-2.02)	4.42	<.001	1.17 (0.96-1.44)	1.55	0.12
Social isolation	2.02 (1.58-2.58)	5.62	<.001	1.44 (1.14-1.81)	3.07	0.002

Odds ratios refer to the group difference between those with persistent externalising and those with no childhood psychopathology, adjusted for sex and socioeconomic factors.

a. Group sizes refers to those included in fully-adjusted model (i.e., with data on all covariates); Lower N available was for educational/economic outcomes analysis (334 females & 419 males with persistent externalising)

© 2023 Dooley N et al. *JAMA Network Open*.

eTable 9. Results of Sex-Stratified Analyses (Internalising Psychopathology)

Fully adjusted effect of childhood internalising on each functional outcome for males and females separately.

	Females			Males		
	104 persistent internalising Vs 1697 no persistent psych. [a]			125 persistent internalising Vs 1630 no persistent psych. [a]		
**Outcome (age 17/20)**	**OR**	**z**	**p**	**OR**	**z**	**p**
Poor mental health	2.59 (1.72-3.90)	4.55	<.001	3.71 (2.52-5.44)	6.68	<.001
Poor subjective wellbeing	1.72 (1.15-2.56)	2.63	0.009	3.80 (2.61-5.53)	6.99	<.001
Poor physical health	3.64 (2.42-5.47)	6.19	<.001	1.52 (0.98-2.35)	1.87	0.06
Substance use	1.71 (1.06-2.77)	2.18	0.03	0.63 (0.36-1.10)	-1.61	0.11
Frequent health service use	1.95 (1.31-2.90)	3.27	0.001	1.01 (0.69-1.49)	0.06	0.95
Social isolation	1.50 (0.94-2.39)	1.72	0.09	2.49 (1.69-3.67)	4.60	<.001
Poor educational/economic outcome	1.53 (0.97-2.42)	1.84	0.07	3.95 (2.53-6.16)	6.05	<.001

Odds ratios refer to the group difference between those with persistent internalising and those with no childhood psychopathology, adjusted for sex and socioeconomic factors.

a. Group sizes refers to those included in fully-adjusted model (i.e., with data on all covariates); Lower N available for educational/economic outcomes analysis (86 females & 103 males with persistent internalising)

© 2023 Dooley N et al. *JAMA Network Open*.

eAppendix. Results of Method Triangulation

We repeated the core analyses using alternative groups of child psychopathology, defined by simple cut-offs on the SDQ scales, rather than LCTA. The 80th percentile was used to identify clinically significant cases of psychopathology (sdqinfo.org)[7,8]. All group sizes and estimates below are weighted to account for drop-off over time and sampling bias.

Definitions of each group are described in the method and eTable 10 below. Of the 6,039 participants in the sampe sample used for the core analyses, just 2,520 (41.7%) met one of these definitions. The 3,519 participants not included are those who were above/below the 80th percentile at one age, and not at the next. Below, eTable 10 shows the breakdown of the 2,520 individuals into each group (weighted by age 13 sample weights); eTable 11 provides average SDQ scores for these groups; and eTables 12-13 provide odds ratios from adjusted analyses.

© 2023 Dooley N et al. *JAMA Network Open*.

eTable 10. Definitions and Group Sizes for the alternative Childhood Psychopathology Groups Used in the Sensitivity Analysis

Group	Definition	N	%
**Persistently Low Psychopathology**	<80th percentile on all SDQ scales at both 9 and 13 years	1,420	56.4%
**Persistent Externalising**	≥80th percentile on either hyperactivity or conduct problems at both 9 and 13 years (<80th percentile on other scales)	544	21.6%
**Persistent Internalising**	≥80th percentile on either peer or emotional problems at both 9 and 13 years (<80th percentile on other scales)	433	17.2%
**Persistently High Psychopathology**	≥80th percentile on all SDQ scales at ages 9 and 13	123	4.9%

© 2023 Dooley N et al. *JAMA Network Open*.

eTable 11. Mean SDQ Scores for the Alternative Persistent Psychopathology Groups Used in the Sensitivity Analysis (n = 2520)

		Mean (SD)			
	**N**	Emotional	Peer	Hyperactivity/ Inattention	Conduct
**Age 9**	2,520				
Low Psychopathology	1,420	0.86 (0.96)	0.27 (0.45)	1.39 (1.27)	0.30 (0.46)
Externalising	544	1.15 (0.99)	0.34 (0.48)	4.93 (2.29)	2.01 (1.35)
Internalising	433	3.23 (2.22)	2.28 (1.35)	1.76 (1.36)	0.39 (0.49)
High Psychopathology	123	6.19 (1.62)	4.56 (2.10)	7.58 (1.85)	4.53 (2.08)
**Age 13**	2,520				
Low Psychopathology	1,420	0.58 (0.75)	0.25 (0.44)	1.00 (1.03)	0.25 (0.44)
Externalising	544	0.79 (0.79)	0.31 (0.46)	4.48 (2.27)	1.81 (1.25)
Internalising	433	2.89 (2.05)	2.18 (1.59)	1.27 (1.11)	0.38 (0.47)
High Psychopathology	123	5.70 (2.08)	3.59 (1.50)	7.31 (1.67)	4.02 (1.84)

© 2023 Dooley N et al. *JAMA Network Open*.

eTable 12. Odds of Adverse Adult Outcomes in Those With Any Persistent Psychopathology From Age 9 to 13 Years Using Alternative Group Definitions for the Sensitivity Analysis

	Unadjusted					Fully Adjusted ±				
	OR	Lower	Upper	z	p	OR	Lower	Upper	z	p
Mental health problems	1.90	1.53	2.36	5.836	<.001	2.06	1.64	2.59	6.194	<.001
Physical health problems	1.79	1.45	2.22	5.432	<.001	2.05	1.63	2.57	6.179	<.001
Heavy substance use	1.65	1.34	2.03	4.654	<.001	1.67	1.33	2.09	4.479	<.001
Frequent health service use	1.25	1.04	1.49	2.407	0.016	1.32	1.10	1.60	2.886	0.004
Social isolation	2.00	1.62	2.47	6.486	<.001	1.86	1.49	2.32	5.529	<.001
Poor educational/economic outcomes	1.72	1.40	2.11	5.143	<.001	1.40	1.12	1.76	2.948	0.003
Poor subjective wellbeing	1.90	1.59	2.28	6.926	<.001	2.23	1.83	2.72	8.057	<.001

± Child's sex, parent education level, single-parenthood & household income at age 13

© 2023 Dooley N et al. *JAMA Network Open*.

eTable 13. Odds Ratios (OR) of Difference Between Childhood Groups in the Odds of Each Adverse Outcome in Young Adulthood (Reference Group: Low Psychopathology)

Both original results (**bold**) and those from the alternative analysis (not bold) are shown. All estimates are adjusted for sex and childhood socioeconomic factors.

Young Adult Outcome	Group Definition Method	OR	Lower	Upper	z	p
Group: **Persistently High Psychopathology**						
Mental Health Problems	**LCTA**	**2.91**	**2.05**	**4.12**	**5.99**	**0.000**
	Cut Point	4.97	3.16	7.81	6.94	0.000
Physical Health Problems	**LCTA**	**1.53**	**1.06**	**2.21**	**2.25**	**0.024**
	Cut Point	1.87	1.15	3.06	2.50	0.012
Heavy substance use	**LCTA**	**2.36**	**1.63**	**3.41**	**4.56**	**0.000**
	Cut Point	1.89	1.17	3.06	2.59	0.010
Frequent health service use	**LCTA**	**1.31**	**0.93**	**1.83**	**1.55**	**0.121**
	Cut Point	1.50	0.97	2.32	1.84	0.065
Social isolation	**LCTA**	**1.79**	**1.23**	**2.59**	**3.06**	**0.002**
	Cut Point	1.79	1.10	2.91	2.34	0.019
Poor subjective wellbeing	**LCTA**	**2.38**	**1.69**	**3.36**	**4.97**	**0.000**
	Cut Point	4.00	2.57	6.22	6.15	0.000
Adverse educational/economic outcomes	**LCTA**	**1.64**	**1.06**	**2.51**	**2.24**	**0.025**
	Cut Point	1.71	0.95	3.07	1.78	0.075
Group: **Persistent Internalising**						
Mental Health Problems	**LCTA**	**3.08**	**2.33**	**4.08**	**7.86**	**0.000**
	Cut Point	2.48	1.88	3.28	6.42	0.000
Physical Health Problems	**LCTA**	**2.31**	**1.74**	**3.07**	**5.75**	**0.000**
	Cut Point	2.91	2.21	3.81	7.69	0.000
Heavy substance use	**LCTA**	**1.04**	**0.73**	**1.50**	**0.23**	**0.819**
	Cut Point	0.96	0.70	1.33	-0.23	0.821
Frequent health service use	**LCTA**	**1.38**	**1.05**	**1.81**	**2.28**	**0.022**
	Cut Point	1.42	1.11	1.82	2.80	0.005
Social isolation	**LCTA**	**1.98**	**1.48**	**2.66**	**4.54**	**0.000**
	Cut Point	1.59	1.20	2.12	3.21	0.001
Poor subjective wellbeing	**LCTA**	**2.62**	**1.98**	**3.46**	**6.79**	**0.000**

© 2023 Dooley N et al. *JAMA Network Open*.

Young Adult Outcome	Group Definition Method	OR	Lower	Upper	z	p
	Cut Point	2.29	1.79	2.93	6.55	0.000
Adverse educational/economic outcomes	**LCTA**	**2.55**	**1.87**	**3.47**	**5.94**	**0.000**
	Cut Point	1.08	0.81	1.44	0.52	0.604
Group: **Persistent Externalising**						
Mental Health Problems	**LCTA**	**1.66**	**1.40**	**1.96**	**5.88**	**0.000**
	Cut Point	1.31	0.96	1.78	1.72	0.086
Physical Health Problems	**LCTA**	**1.53**	**1.30**	**1.80**	**5.02**	**0.000**
	Cut Point	1.38	1.02	1.87	2.09	0.036
Heavy substance use	**LCTA**	**1.77**	**1.49**	**2.11**	**6.44**	**0.000**
	Cut Point	2.42	1.86	3.15	6.55	0.000
Frequent health service use	**LCTA**	**1.38**	**1.19**	**1.60**	**4.32**	**0.000**
	Cut Point	1.20	0.94	1.54	1.48	0.139
Social isolation	**LCTA**	**1.65**	**1.40**	**1.95**	**5.86**	**0.000**
	Cut Point	2.15	1.64	2.80	5.61	0.000
Poor subjective wellbeing	**LCTA**	**1.80**	**1.55**	**2.09**	**7.65**	**0.000**
	Cut Point	1.92	1.50	2.46	5.17	0.000
Adverse educational/economic outcomes	**LCTA**	**1.98**	**1.67**	**2.35**	**7.83**	**0.000**
	Cut Point	1.76	1.32	2.33	3.89	0.000

LCTA = Latent Class Transition Analysis (as defined in [9])

© 2023 Dooley N et al. *JAMA Network Open*.

eFigure 3. Adjusted Odds of Poor Young Adult Outcomes for Childhood Mental Health Groups Used in the Sensitivity Analysis

Reference group: persistently low childhood psychopathology. Bold ORs refer to those significant at Bonferroni-corrected level.

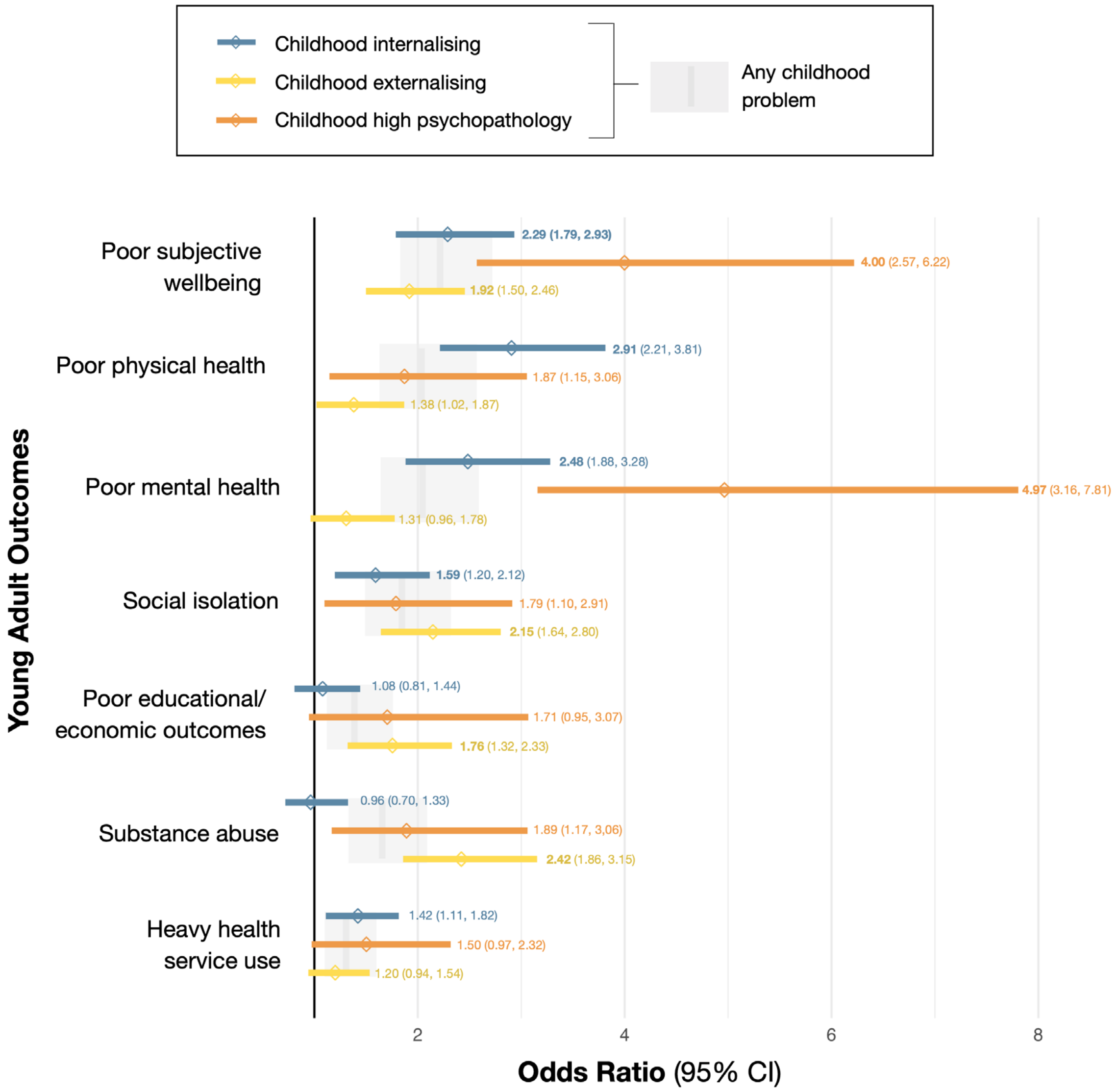

© 2023 Dooley N et al. *JAMA Network Open*.

eReferences.

1. Babor TF, Higgins-Biddle JC, Saunders JB, Monteiro MG. *The alcohol use disorders identification test*. World Health Organization Geneva; 2001.

2. Brugha TS, Weich S, Singleton N, et al. Primary group size, social support, gender and future mental health status in a prospective study of people living in private households throughout Great Britain. *Psychol Med*. 2005;35(5):705-14.

3. Saini P, McIntyre J, Corcoran R, et al. Predictors of emergency department and GP use among patients with mental health conditions: a public health survey. *Br J Gen Pract*. 2020;70(690):e1-e8.

4. O'Mahony D, McNamara E, McClintock R, Murray A, Smyth E, Watson D. *The Lives of 20-year-olds: making the transition to adulthood*. Growing Up in Ireland; 2021.

5. Greene S, Williams J, Layte R, et al. Growing up in Ireland National Longitudinal Study of Children Background and Conceptual Framework. 2010;

6. Murphy D, Quail A, Williams J, et al. *A summary guide to wave 3 of Growing Up in Ireland's child cohort (at 17/18 years)*. 2020. https://www.growingup.gov.ie/growing-up-in-ireland-publications/

7. Goodman R, Meltzer H, Bailey V. The Strengths and Difficulties Questionnaire: a pilot study on the validity of the self-report version. *Int Rev Psychiatry*. 2003;15(1-2):173-177.

8. He J-P, Burstein M, Schmitz A, Merikangas KR. The Strengths and Difficulties Questionnaire (SDQ): the Factor Structure and Scale Validation in U.S. Adolescents. *J Abnorm Child Psychol*. 2013;41(4):583-595.

9. Healy C, Brannigan R, Dooley N, et al. Person-Centered Trajectories of Psychopathology From Early Childhood to Late Adolescence. *JAMA Netw Open*. 2022;5(5):e229601.

© 2023 Dooley N et al. *JAMA Network Open*.
