# Supplementary material for: Functional Outcomes Among Young People With Trajectories of Persistent Childhood Psychopathology
Source: JAMA Netw Open. 2023 Sep 29;6(9):e2336520. doi: 10.1001/jamanetworkopen.2023.36520 (PMC10543080; doi:10.1001/jamanetworkopen.2023.36520)
Supplement: Supplement 2. — Data Sharing Statement [file jamanetwopen-e2336520-s002.pdf]

Data Sharing Statement

Dooley. Functional Outcomes Among Young People With Trajectories of Persistent Childhood Psychopathology. *JAMA Netw Open*. Published September 29, 2023.
doi:10.1001/jamanetworkopen.2023.36520

Data
Data available: Yes
Data types: Deidentified participant data, Data dictionary
How to access data: https://www.ucd.ie/issda/data/guichild/
When available: beginning date: 01-01-2019

Supporting Documents
Document types: None

Additional Information
Who can access the data: Researchers affiliated with an Irish institution
Types of analyses: For teaching & research purposes
Mechanisms of data availability: Approval of a data request form
